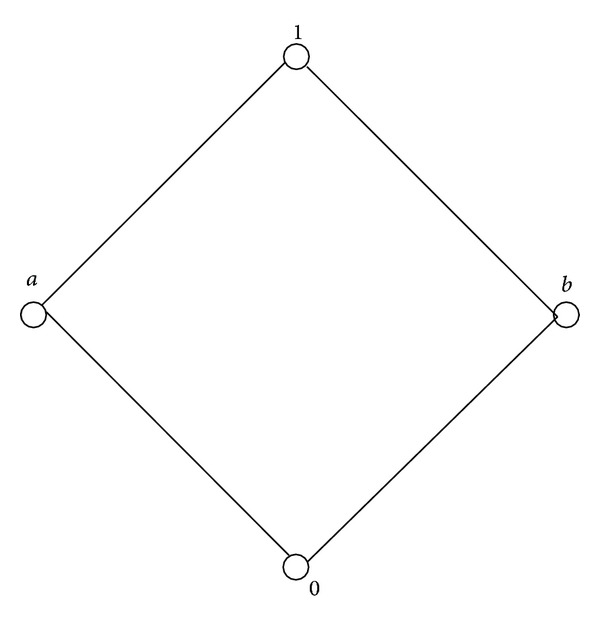# Roughness in Lattice Ordered Effect Algebras

**DOI:** 10.1155/2014/542846

**Published:** 2014-07-24

**Authors:** Xiao Long Xin, Xiu Juan Hua, Xi Zhu

**Affiliations:** ^1^Department of Mathematics, Northwest University, Xi'an 710127, China; ^2^Faculty of Science, Xi'an University of Technology, Xi'an 710048, China; ^3^Department of Basic Courses, Xi'an Aeronautical University, Xi'an 710048, China

## Abstract

Many authors have studied roughness on various algebraic systems. In this paper, we consider a lattice ordered effect algebra and discuss its roughness in this context. Moreover, we introduce the notions of the interior and the closure of a subset and give some of their properties in effect algebras. Finally, we use a Riesz ideal induced congruence and define a function *e*(*a*, *b*) in a lattice ordered effect algebra *E* and build a relationship between it and congruence classes. Then we study some properties about approximation of lattice ordered effect algebras.

## 1. Introduction

Quantum effects play a basic role in the foundations of quantum mechanics. In 1994, Foulis and Bennett introduced effect algebras for modeling unsharp measurement in a quantum mechanical system [1]. In the same year, Kôpka and Chovanec introduced equivalent structures called D-posets [2]. It is a generalization of many structures which arise in quantum physics [3] and in mathematical economics [4, 5], in particular, of orthomodular lattices in noncommutative measure theory and MV-algebras in fuzzy measure theory. Effect algebras were originally introduced as partial algebraic structures and after 1994, there have been a great number of papers concerning effect algebras [6–11]. They are a generalization of many structures which arise in quantum physics.

The theory of rough sets was first introduced by Pawlak [12] as a tool for dealing with granularity in knowledge. Rough set theory, a new mathematical approach to deal with inexact, uncertain, or vague knowledge, has recently received wide attention on the research areas in both of the real life applications and the theory itself. Rough set theory assumes that every object in a universe of discourse is linked with some form of characterizations. Objects described using the same characterizations are indiscernible. An elementary set in a rough set theory consists of all indiscernible objects and forms the smallest piece of knowledge about the universe. All the elementary sets form a partition of the universe. Any set formed from a union of elementary sets is called an observable, definable, or crisp set. Otherwise, it is called a rough (vague) set. Due to the granularity of knowledge, rough sets cannot be characterized by using available knowledge. Therefore with every rough set we associate two crisp sets, called its lower and upper approximation. Intuitively, the lower approximation of a set consists of all elements that surely belong to the set, whereas the upper approximation of the set constitutes all elements that possibly belong to the set. The difference of the upper and the lower approximation is a boundary region. It consists of all elements that cannot be classified uniquely to the set or its complement, by employing available knowledge. Thus any rough set, in contrast to a crisp set, has a nonempty boundary region. The lower and upper approximations form the most precise approximations of the given by crisp sets. It is well known that a partition induces an equivalence relation on a universe and vice versa. The properties of rough sets can thus be examined via either partitions or equivalence classes. Rough set theory is emerging as a powerful theory dealing with imperfect data. It is an expending research area which stimulates explorations on both real world applications and on the theory itself. It has found practical applications in many areas such as knowledge discovery, data analysis, approximate classification, and conflict analysis. The reader will find in [4, 5, 8, 12–25] the deep study of rough set theory. It soon invoked a natural question concerning possible connection between rough sets and algebras. Davvaz discussed the roughness of ring [4], Hv-group [13], Hv-module [14], hyperring [15], and so on. In [26], as a generalization of ideals in BCK-algebras, the notion of rough ideals is discussed.

Events of quantum logics do not describe “unsharp measurements” since unsharp measurements do not have a “yes-no” character. To include such events another algebraic structure was introduced by Foulis and Bennett (1994), called an effect algebra. Hence elements of an effect algebra *E* can be regarded as (possibly) unsharp experimentally testable propositions about a physical system. A subset *X* of *E* can be regarded as (possibly) some unsharp experimentally testable propositions about a physical system. We think about a rough approximation of *X* to be the set of propositions which is indiscernible with some propositions in *X*. It means that two propositions *p* and *q* are observable simultaneously in terms of quantum measurement theory that *p*, *q* ∈ *E* are indiscernible.

In this paper, we discuss the roughness of lattice ordered effect algebra and introduce the notions of the interior and the closure of a subset. We give some properties of the interior and the closure of a subset in lattice ordered effect algebras.

## 2. Basic Concepts and Properties of Effect Algebras and Approximation Spaces

### 2.1. Effect Algebras

In this section, we recall some definitions and results which will be used in the sequel.

**Definition 1 (Foulis and Bennett [1]).**

An effect algebra is a partial algebra *E* with partial binary operation ⊕ and two nullary operations 0 and 1 satisfying the following axioms.

(E1) *a* ⊕ *b* = *b* ⊕ *a* if *a* ⊕ *b* is defined.
(E2) (*a* ⊕ *b*) ⊕ *c* = *a* ⊕ (*b* ⊕ *c*) if one side is defined.
(E3) For every *a* ∈ *E* there exists a unique *b* ∈ *E* such that *a* ⊕ *b* = 1.
(E4) If 1 ⊕ *a* is defined then *a* = 0.

**Example 2 (Gudder [27]).**

(1) A simple example of an effect algebra is [0,1]⊆*R*, where *a*⊥*b* is defined by *a* + *b* ≤ 1, in which case *a* ⊕ *b* = *a* + *b*.

(2) Another example is an *n*-chain, *C*_*n*_ = {0, *a*, 2*a*,…, *na* = 1}, where *ia*⊥*ja* if and only if *i* + *j* ≤ *n* for *i*, *j* = 0,1, 2,…, *n*, in which case *ia* ⊕ *ja* = (*i* + *j*)*a*.

(3) Let *H* be a complex Hilbert space and let Φ(*H*) be the set of all bounded self-adjoint operators on *H*. The positive cone Φ(*H*)^+^ in Φ(*H*) is the set of all *A* ∈ Φ(*H*) that satisfy 〈*Ax*, *x*〉 ≥ 0 for all *x* ∈ *H*. We then write *A* ≤ *B* if *B* − *A* ∈ Φ(*H*)^+^. Letting 0 and 1 be the zero and identity operators, respectively, we have that 1 ∈ Φ(*H*)^+^ and (Φ(*H*), +, 0, ≤) is a partially ordered Abelian group. It can be checked that *℘*(*H*) = Φ(*H*)^+^[0,1] is an effect algebra but not a Boolean effect algebra.

Having an effect algebra *E*, we can introduce a partial order ≤ on *E*: *a* ≤ *b* iff there exists *c* : *a* ⊕ *c* = *b*. We denote *b* ⊖ *a* = *c* iff *a* ⊕ *c* = *b*. It is easy to check that ⊖ is a well defined partial operation. In [7], a class of partial structures equivalent to effect algebra, so-called D-posets, was introduced independently. The axioms for D-posets are based on ⊖.

As usual, we denote 1 ⊖ *x* by *x*′. Further, we denote *a*⊥*b* iff *a* ⊕ *b* exists iff *a* ≤ *b*′ iff *b* ≤ *a*′. If *E* is an effect algebra and (*E*, ≤) is a lattice, then *E* is called lattice ordered and it is said to be distributive iff, as a poset, it forms a distributive lattice. Every lattice ordered effect algebra satisfies the De Morgan law: (*a*∨*b*)′ = *a*′∧*b*′. Lattice ordered effect algebras are called D-lattices in [2]. If (*E*, ≤, 0,1) is a bounded lattice with the orthosupplement operation ′ satisfying the orthomodular law: for all *a*, *b* ∈ *E*, *a* ≤ *b*⇒*b* = *a*∨(*a*∨*b*′)′, then (*E*, ≤) is said to be orthomodular lattice.

**Lemma 3 (Foulis and Bennett [1]).**

Let *E* be an effect algebra, *a*, *b* ∈ *E*.

1. *a*⊥*b*⇒*b*⊥*a*,
2. *a*′′ = *a*,
3. 1′ = 0* and *0′ = 1,
4. *a* ⊕ *a*′ = 1,
5. *a*⊥0* and a* ⊕ 0 = *a*,
6. *a*⊥1⇔*a* = 0,
7. *a* ⊕ *b* = 0⇒*a* = *b* = 0.
8. *a*⊥*b*⇒*a*⊥(*a* ⊕ *b*)′* and a* ⊕ (*a* ⊕ *b*)′ = *b*′,
9. *a*⊥*b*⇔*a* ≤ *b*′,
10. *a* ≤ *b*⇒*b*′ ≤ *a*′,
11. *a* ≤ *b*⇒*a*⊥(*a* ⊕ *b*′)′ and *a* ⊕ (*a* ⊕ *b*′)′ = *b*.

**Lemma 4 (Foulis and Bennett [1]).**

Let *E* be an effect algebra. If *a*, *b* ∈ *E* with *a* ≤ *b*, then

- *a* = *b*⇔*b* ⊖ *a* = 0,
- *a* = 0⇔*b* ⊖ *a* = *b*,
- *b* ⊖ *a* ≤ *b* *and a* = *b* ⊖ (*b* ⊖ *a*),
- *c* ≤ *b* ⊖ *a*⇒*a* ≤ *b* ⊖ *c and *(*b* ⊖ *a*) ⊖ *c* = (*b* ⊖ *c*) ⊖ *a*,
- *b* ⊖ *a* = (*a* ⊕ *b*′)′,
- *a* ⊕ *b*′ = (*b* ⊖ *a*)′.

**Lemma 5 (Foulis and Bennett [1]).**

Let *E* be an effect algebra. If *p*, *q*, *r* ∈ *E* and *p* ≤ *q* ≤ *r*, then the following statements hold:

- *r* = *p* ⊕ (*q* ⊖ *p*)⊕(*r* ⊖ *q*),
- *r* ⊖ *p* = (*q* ⊖ *p*)⊕(*r* ⊖ *q*),
- (*r* ⊖ *p*)⊖(*r* ⊖ *q*) = *q* ⊖ *p*,
- *r* ⊖ (*q* ⊖ *p*) = *p* ⊕ (*r* ⊖ *q*).

**Lemma 6 (Riečanová [28]).**

In an effect algebra (*E*, ⊕, 0,1), the following statements are satisfied.

- * For all a*, *b*, *u*, *v* ∈ *E, if u* ≤ *a*, *v* ≤ *b, and a*⊥*b, then u* ⊕ *v is defined.*
- * For all a*, *b*, *c* ∈ *E, if b*⊥*c, then a* ≤ *b if and only if a* ⊕ *c* ≤ *b* ⊕ *c*.
- * For all a*, *b* ∈ *E*, *a* ≤ *b if and only if b*′ ≤ *a*′.

*Moreover, if E* *is lattice ordered, for all a*, *b*, *c* ∈ *E, we have the following.*

(iv) * If a*⊥*c and* *b*⊥*c, then *(*a* ⊕ *c*)∨(*b* ⊕ *c*) = (*a*∨*b*) ⊕ *c*.
(v) * If a* ≥ *c*, *b* ≥ *c, then *(*a* ⊖ *c*)∨(*b* ⊖ *c*) = (*a*∨*b*) ⊖ *c*.

**Definition 7 (Foulis and Bennett [1]).**

A subset *Q* of an effect algebra (*E*, ⊕, 0,1) is called a subeffect algebra of *E* if and only if 0,1 ∈ *Q*, *Q* is closed under *p* ↦ *p*′, and for all *p*, *q* ∈ *Q*, *p*⊥*q*⇒*p* ⊕ *q* ∈ *Q*.

**Definition 8 (Gudder [29]).**

Let (*E*, ⊕, 0,1) be an effect algebra; an element *a* ∈ *E* is called sharp if *a*∧*a*′ = 0. The set of all sharp elements is denoted by *E*_*S*_.

In [29] it has been shown that in a lattice ordered effect algebra *E*, *E*_*S*_ is a subeffect algebra that is an orthomodular lattice. Obviously, in an effect algebra *E*, 0,1 ∈ *E*_*S*_.

**Definition 9 (Greechie et al. [30]).**

Let *E* be an effect algebra. If *e* ∈ *E*, for *p*, *q* ∈ *E*, *p*⊥*q* and *p*, *q* ≤ *e*⇒*p* ⊕ *q* ≤ *e*, then *e* is called principal.

**Definition 10 (Greechie et al. [30]).**

An element *a* of an effect algebra *E* is called central, if

1. *a* and *a*′ are principal,
2. for every *b* ∈ *E* there are *b*_1_, *b*_2_ ∈ *E* such that *b*_1_ ≤ *a*, *b*_2_ ≤ *a*′ and *b* = *b*_1_ ⊕ *b*_2_.

The center *C*(*E*) of the effect algebra *E* is the set of all central elements of *E*. *C*(*E*) is a subeffect algebra of the effect algebra *E* and forms a Boolean algebra (see [30]).

**Definition 11 (Ma [10]).**

Let (*E*, ⊕, 0,1) be an effect algebra. A nonempty subset *I* of (*E*, ⊕, 0,1) is said to be an effect algebra ideal of (*E*, ⊕, 0,1), if the following conditions are satisfied: for all *a*, *b* ∈ *E*,

- *a* ∈ *I*, *b* ≤ *a* implies *b* ∈ *I*,
- *a* ⊖ *b* ∈ *I*, *b* ∈ *I* implies *a* ∈ *I*.

Equivalently, a nonempty subset *I* of the effect algebra (*E*, ⊕, 0,1) is an effect algebra ideal iff it satisfies the condition that if *a* ⊕ *b* is defined, then *a* ⊕ *b* ∈ *I*⇔*a*, *b* ∈ *I*.

**Definition 12 (Jenca and Pulmannová [9]).**

Let (*E*, ⊕, 0,1) be an effect algebra and let *I* be an ideal *I* of *E*. If for any *i* ∈ *I*, *a*, *b* ∈ *E*, *a*⊥*b*, *i* ≤ *a* ⊕ *b* implies that there are *a*_1_, *b*_1_ ∈ *I*, *a*_1_ ≤ *a*, *b*_1_ ≤ *b* with *i* ≤ *a*_1_ ⊕ *b*_1_, one calls *I* a Riesz ideal.

**Definition 13 (Jenca and Pulmannová [9]).**

Let (*E*, ⊕, 0,1) be an effect algebra and let *I* be an ideal *I* of *E*. Define a binary relation ~_*I*_ on *E* by *a* ~ _*I*_ *b* if and only if there are *i*, *j* ∈ *I*, *i* ≤ *a*, *j* ≤ *b* such that *a* ⊖ *i* = *b* ⊖ *j*.

Equivalently, *a* ~ _*I*_ *b* if and only if there is *k* ∈ *E*, *k* ≤ *a*, *b* and *a* ⊖ *k*, *b* ⊖ *k* ∈ *I*.

In particular, for each *a* ∈ *E* we have *a* ~ _*I*_0 if and only if *a* ∈ *I*.

**Definition 14 (Jenca and Pulmannová [9]).**

A binary relation ~ on an effect algebra *E* is called a congruence relation if the following conditions are satisfied:

- ~ is an equivalence relation,
- *a* ~ *a*_1_, *b* ~ *b*_1_, *a*⊥*b*, *a*_1_⊥*b*_1_ implies *a* ⊕ *b* ~ *a*_1_ ⊕ *b*_1_,
- if *a* ~ *b*, *b*⊥*c* then there exists *d* ∈ *E* such that *d* ~ *c* and *a*⊥*d*.

**Lemma 15 (Jenca and Pulmannová [9]).**

Let *I* be an ideal in an effect algebra *E*. Then ~_*I*_ is a congruence if and only if *I* is a Riesz ideal.

This means that *a* ~ _*I*_ *b* implies *a* ⊕ *c* ~ _*I*_ *b* ⊕ *c*. The equivalence class of *a* ∈ *E* is denoted by [*a*]_*I*_. Note that if *I*, *J* are Riesz ideals and *I*⊆*J*, then [*a*]_*I*_⊆[*a*]_*J*_.

**Definition 16 (Foulis and Bennett [1]).**

Let *E*, *F* be effect algebras. A mapping *h* : *E* → *F* is said to be

- a morphism iff *h*(1) = 1 and *a*, *b* ∈ *E* with *a*⊥*b*⇒*h*(*a*)⊥*h*(*b*) and *h*(*a* ⊕ *b*) = *h*(*a*) ⊕ *h*(*b*);
- a homomorphism iff *h* is a morphism and *a*, *b* ∈ *E* with *a*∧*b* = 0⇒*h*(*a*)∧*h*(*b*) = 0;
- a monomorphism iff *h* is a morphism and *a*, *b* ∈ *E* with *h*(*a*) ≤ *h*(*b*)⇒*a* ≤ *b*.

A morphism *h* : *E* → *F* is called a ∧-morphism if *h*(*a*∧*b*) = *h*(*a*)∧*h*(*b*) whenever *a*∧*b* exists in *E*. A morphism *h* such that *a*⊥*b* iff *h*(*a*)⊥*h*(*b*) is a monomorphism. It is clear that *h*(0) = 0 and that for *a*, *b* ∈ *E*, *a* ≤ *b*⇒*h*(*a*) ≤ *h*(*b*) with *h*(*b* ⊖ *a*) = *h*(*b*) ⊖ *h*(*a*). In particular, *h*(*a*′) = *h*(*a*)′ (see [1]).

**Lemma 17 (Ma [10]).**

If *E* is an effect algebra in which every element is principle, then for all *x*, *y* ∈ *E*, *x*⊥*y* implies that *x* ⊕ *y* = *x*∨*y*.

**Remark 18 .**

An MV-algebra is the same thing as a lattice ordered effect algebra in which disjoint pairs and a Boolean algebra is the same thing as a distributive lattice ordered effect algebra in which orthogonal pairs are disjoint pairs. Also, every MV-algebra is distributive.

### 2.2. Approximation Spaces

Let *U* denote a finite set and let *θ*⊆*U* × *U* be an indiscernibility relation on *U*. Apr is defined to be the pair (*U*, *θ*) and is called an approximation space. Intuitively, elements of a *θ*-equivalence class are to be regarded as indiscernible from one another.

**Definition 19 .**

For an approximation space (*U*, *θ*), by a rough approximation in (*U*, *θ*) one means a mapping Apr : *P*(*U*) → *P*(*U*) × *P*(*U*) defined for every *X* ∈ *P*(*U*) by $\text{Apr}(X)=(X^{0},\bar{X})$ where $\bar{X}=\{x\in U:{\left[x\right]}_{\theta }\cap X\neq \emptyset \}$, *X* ^0^ = {*x* ∈ *U* : [*x*]_*θ*_⊆*X*}. Here *X* ^0^ is called the interior (or the lower rough approximation) of *X*, $\bar{X}$ is called the closure (or the upper rough approximation) of *X*, and $\bar{X}\setminus X^{0}$ is called the boundary of *X*. Apr(*X*) is called the rough approximation of *X* (or the rough set) in (*U*, *θ*) (see [4, 5, 13–17, 19–21, 23–25, 31, 32]).

For the sake of illustration, we give the following example (see [4]).

**Example 20 .**

Let (*U*, *θ*) be an approximation space, where *U* = {*x*_1_, *x*_2_,…, *x*_8_} and let *θ* be an equivalence relation with the following equivalence classes:

(1) E1={x1,x4,x8},  E2={x2,x5,x7},E3={x3},  E4={x6}.

Let *X* = {*x*_3_, *x*_5_}; then *X* ^0^ = {*x*_3_} and $\bar{X}=\;\{x_{2},x_{3},x_{5},x_{7}\}$ and so ({*x*_3_}, {*x*_2_, *x*_3_, *x*_5_, *x*_7_}) = Apr(*X*) is a rough set.

Now, we give an example of the lower and upper approximation theory applied to effect algebras.

**Example 21 .**

Let *E* consist of the six distinct elements 0, *p*, *q*, *p*′, *q*′, 1, where *p*′ and *q*′ are the only nonzero elements which are orthogonal to *p* and *q*, respectively. Furthermore, let *p* ⊕ *p*′ = *q* ⊕ *q*′ = 1. See the following table. Then *E* is an effect algebra. Denote by *I* the set {0, *p*, *q*} and by *θ* the equivalence relation defined as follows:

∀*a*, *b* ∈ *E*, [*aθb*⇔ either *a*, *b* ∈ *I* or *a*, *b* ∉ *I*],

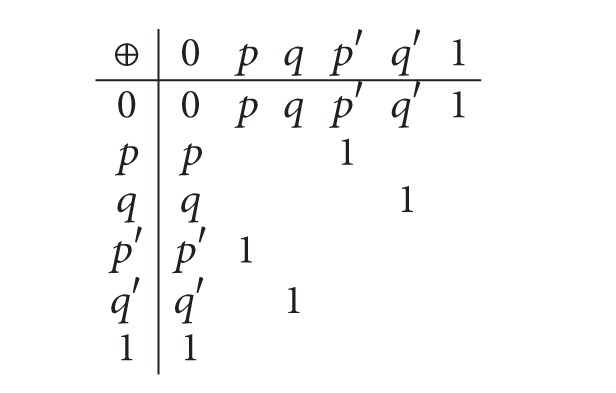
(2)


Clearly *θ* is a congruence and [0]_*θ*_ = [*p*]_*θ*_ = [*q*]_*θ*_ = *I* and [*p*′]_*θ*_ = [*q*′]_*θ*_ = [1]_*θ*_ = {*p*′, *q*′, 1}. Let *X* = {0,1}; then *X* ^0^ = *∅* and $\bar{X}=E$.

Let (*U*, *θ*) be an approximation space and let *X* be a subset of *U*.

- If $X^{0}=\bar{X}$, then *X* is called a crisp set.
- If *X* ^0^ = *∅*, then *X* is called having an empty interior with respect to *θ*.
- We define $U\setminus \bar{X}$ to be the exterior of *X*. If $\bar{X}=U$, then *X* is called having an empty exterior with respect to *θ*.

Let (*U*, *θ*) be an approximation space. Clearly, *∅* and *U* are definable with respect to *θ*. The family of all crisp sets is denoted by Cri(Apr).

**Example 22 .**

In Example 20, *X* is not a crisp set, has no empty interior, and has no empty exterior with respect to *θ*. In Example 21, *X* is not a crisp set, but it has empty interior and empty exterior with respect to *θ*.

## 3. Approximation in Effect Algebras

In this section, we present some properties of approximation spaces generally and approximation in effect algebras.

Let *E* be an effect algebra, and let *I* be a Riesz ideal of *E* and let *X* be a subset of *E*. Then the sets *X*_*I*_^0^ = {*x* ∈ *E* : [*x*]_*I*_⊆*X*}, $\bar{X_{I}}=\{x\in E:{\left[x\right]}_{I}\cap X\neq \emptyset \}$ are called, respectively, the interior and closure (or the lower and upper approximations) of the set *X* with respect to the ideal *I*. When *U* = *E* and *θ* is the equivalence relation defined in Definition 14, then we use the pair (*E*, *I*) instead of the approximation space (*U*, *θ*). Also, in this case we use the symbols $\bar{X_{I}}$ and *X*_*I*_^0^ instead of $\bar{X}$ and *X* ^0^.

**Lemma 23 .**

Let *E* be an effect algebra, let *I* be a Riesz ideal of *E*, and let *X* be a subset of *E*. Then the following statements are equivalent:

1. *X* is a crisp set;
2. *X*_*I*_^0^ = *X* iff $\bar{X_{I}}=X$;
3. *X* is a union of some equivalence classes.

**Proof**

The proof is trivial.

The following properties of approximation spaces are well known and obvious. They are similar to Proposition  3.1 in [4] and Proposition  3.1.1 in [22].

**Proposition 24 .**

Let *E* be an effect algebra and let *I* be a Riesz ideal of *E*. For the approximation space (*E*, *I*), for arbitrary subsets *X*, *Y*⊆*E*, and for each *x* ∈ *X*, one has

1. $X_{I}^{0}\subseteq X\subseteq \bar{X_{I}}$,
2. *E* and *∅* are crisp sets,
3. *X*_*I*_^0^, $\bar{X_{I}}$, and [*X*]_*I*_ are crisp sets with respect to *I*,
4. if *X*⊆*Y*, then *X*_*I*_^0^⊆*Y*_*I*_^0^ and $\bar{X_{I}}\subseteq \bar{Y_{I}}$,
5. $X_{I}^{0}={\left(\bar{X_{I}^{c}}\right)}^{c}$,
6. $\bar{X_{I}}={\left({\left(X^{c}\right)}_{I}^{0}\right)}^{c}$,
7. (*X*∩*Y*)_*I*_^0^ = *X*_*I*_^0^∩*Y*_*I*_^0^,
8. $\bar{{\left(X\cap Y\right)}_{I}}\subseteq \bar{X_{I}}\cap \bar{Y_{I}}$,
9. (*X* ∪ *Y*)_*I*_^0^⊇*X*_*I*_^0^ ∪ *Y*_*I*_^0^,
10. $\bar{{\left(X\cup Y\right)}_{I}}=\bar{X_{I}}\cup \bar{Y_{I}}$,
11. *X*_*E*_^0^ = *∅*, $\bar{X_{E}}=E$,
12. *X*_{0}_^0^ = *X*, $\bar{X_{\left\{0\right\}}}=X$.

The rough complement of Apr(*X*) denoted by Apr^*c*^(*X*) is defined by ${\text{Apr}}^{c}(X)=(U\setminus \bar{X},U\setminus X^{0})$.

**Proof**

The proof is similar to the [31, Theorem  2.1] and [16, Proposition  4.1].

The following example shows that the equations in (8) and (9) of the Proposition 24 do not hold.

**Example 25 .**

Let *E* = {0, *a*, *b*, 1}. Consider the following table:

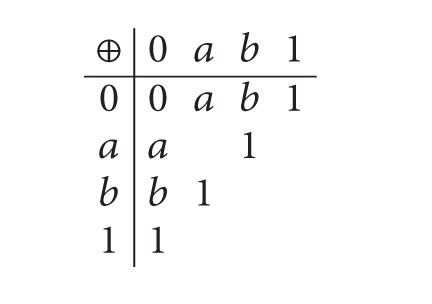
(3)

Then (*E*; ⊕, 0,1) is a Boolean effect algebra with the order given by Figure 1. So (*E*; ⊕, 0,1) is also a lattice ordered effect algebra. Now, we also can check that *I* = {0, *a*} is a Riesz ideal. Let *X* = {*a*, 1} and *Y* = {0, *b*, 1} be subsets of *E*. Then the equivalence classes are [0]_*I*_ = [*a*]_*I*_ = {0, *a*}, [*b*]_*I*_ = [1]_*I*_ = {*b*, 1}. Therefore, we have *X*_*I*_^0^ = *∅*, *Y*_*I*_^0^ = {*b*, 1}, $\bar{X_{I}}=E$, $\bar{Y_{I}}=E$, (*X* ∪ *Y*)_*I*_^0^ = *E*, $\bar{{\left(X\cap Y\right)}_{I}}=\{b,1\}$, and so (*X* ∪ *Y*)_*I*_^0^ ⊈ *X*_*I*_^0^ ∪ *Y*_*I*_^0^, $\bar{X_{I}}\cap \bar{Y_{I}}\;⊈\;\bar{{\left(X\cap Y\right)}_{I}}$.

**Example 26 .**

Let *E* = {0, *a*, *b*, *c*, *d*, *e*, 1}. Define *a* ⊕ *b* = *b* ⊕ *a* = *c*, *b* ⊕ *c* = *c* ⊕ *b* = *a* ⊕ *d* = *d* ⊕ *a* = *e* ⊕ *e* = 1, and *x* ⊕ 0 = 0 ⊕ *x* for all *x* ∈ *E*. We can easily see that *E* is a lattice effect algebra (which is not Boolean). We also can check that *I* = {0, *a*} is a Riesz ideal. Let *X* = {*a*, 1} and *Y* = {0, *b*, *c*} be subsets of *E*. Then [0]_*I*_ = [*a*]_*I*_ = {0, *a*}, [*b*]_*I*_ = [*c*]_*I*_ = {*b*, *c*}, [*d*]_*I*_ = [1]_*I*_ = {*d*, 1}, [*e*]_*I*_ = {*e*}. Therefore, we have $\bar{X_{I}}=\emptyset$, *Y*_*I*_^0^ = {*b*, *c*}, $\bar{X_{I}}=\{0,a,d,1\}$, $\bar{Y_{I}}=\{0,a,b,c\}$, (*X* ∪ *Y*)_*I*_^0^ = {0, *a*, *b*, *c*}, $\bar{{\left(X\cap Y\right)}_{I}}=\emptyset$, and so (*X* ∪ *Y*)_*I*_^0^ ⊈ *X*_*I*_^0^ ∪ *Y*_*I*_^0^, $\bar{X_{I}}\cap \bar{Y_{I}}\;⊈\;\bar{{\left(X\cap Y\right)}_{I}}$.

Let *X* be a subset of an effect algebra *E* and let *X* ^∧^ be the annihilator of *X* in *E* defined by *X* ^∧^ = {*a* ∈ *E* : *a*∧*x* = 0, ∀*x* ∈ *X*}. Denote *X* ^⊥^ = (*X*′)^∧^, where *X*′ = {*x*′ ∈ *E* : *x* ∈ *X*}.

**Proposition 27 .**

Let *I* be a Riesz ideal of effect algebra *E* and let *X* be a subset of *E*. Then

1. (*X* ^⊥^)_*I*_^0^ ⊆ (*X*_*I*_^0^)^⊥^,
2. ${\bar{X_{I}}}^{⊥}\;\subseteq \;\bar{X_{I}^{⊥}}$,
3. ${\bar{X_{I}}}^{⊥}\;\subseteq \;{\left(X_{I}^{0}\right)}^{⊥}$,
4. ${\left(X^{⊥}\right)}_{I}^{0}\;\subseteq \;\bar{{\left(X^{⊥}\right)}_{I}}$.

**Proof**

(1) By Proposition 24, we have *X*_*I*_^0^⊆*X*. It is easy to see that if *X*⊆*Y* then *Y* ^⊥^⊆*X* ^⊥^, so we can take *X* ^⊥^⊆(*X*_*I*_^0^)^⊥^ and again by Proposition 24, we have (*X* ^⊥^)_*I*_^0^⊆(*X*_*I*_^0^)^⊥^. Similarly, we can prove (2), (3), and (4).

The following example shows that inclusion symbols ⊆ in Proposition 27 may not be replaced by equal sign.

**Example 28 .**

Let (*E*; ⊕, 0,1) be a lattice ordered effect algebra in Example 25. Let *X* = {*a*}, *Y* = {0, *a*} be subsets of *E* and let *I* = {0, *a*} be a Riesz ideal of *E*. It is easy to check that *X* ^⊥^ = {0, *a*}, *Y* ^⊥^ = {0}, [0]_*I*_ = [*a*]_*I*_ = *I*, [1]_*I*_ = [*b*]_*I*_ = {*b*, 1}. So we have (*Y* ^⊥^)_*I*_^0^ = *∅*, (*Y*_*I*_^0^)^⊥^ = {0}, ${\left(\bar{Y_{I}}\right)}^{⊥}=\{0\}$, ${\bar{\left(Y^{⊥}\right)}}_{I}=\{0,a\}$. Hence,

 (*Y*_*I*_^0^)^⊥^ ⊈ (*Y* ^⊥^)_*I*_^0^,
 
$\bar{{\left(Y^{⊥}\right)}_{I}}\;⊈\;{\bar{Y_{I}}}^{⊥}$,
 
$\bar{{\left(Y^{⊥}\right)}_{I}}\;⊈\;{\left(Y^{⊥}\right)}_{I}^{0}$.

**Lemma 29 .**

Let *I*, *J* be two Riesz ideals of effect algebra *E* such that *I*⊆*J* and let *X* be a subset of *E*. Then

- *X*_*J*_^0^⊆*X*_*I*_^0^,
- $\bar{X_{I}}\subseteq \bar{X_{J}}$.

**Proof**

It is straightforward.

## 4. Approximation in Lattice Ordered Effect Algebras

In this section, we define a function *e*(*a*, *b*) in a lattice ordered effect algebra *E* and build a relationship between it and congruence classes. Then we study some properties about approximation in lattice ordered effect algebras.

We give some examples of lattice ordered effect algebras.

**Example 30 .**

(1) In Example 2 (1), [0,1]⊆*R* is also a lattice ordered effect algebra.

(2) Let *G* be a partially ordered abelian group with an operator “+”. Let *u* ∈ *G* with *u* > 0, and let *L* : = *G* ^+^[0, *u*] = {*g* ∈ *G*∣0 ≤ *g* ≤ *u*}. Then *L* can be organized into a lattice ordered effect algebra (*L*, 0, *u*, ⊕) by defining *p* ⊕ *q* iff *p* + *q* ≤ *u*, in which case *p* ⊕ *q* = *p* + *q*. In the effect algebra *L* we have *a*′ = *u* − *a* and the effect algebra partial order on *L* coincides with the restriction to *L* of the partial order on *G*. An effect algebra of the form *G* ^+^[0, *u*] (or isomorphic to an effect algebra of this form) is called an interval effect algebra or, for short, an interval algebra.

(3) Effect algebra *E* in Example 25 is a lattice ordered effect algebra.

In a lattice ordered effect algebra *E*, we define *e*(*a*, *b*) = (*a* ⊖ (*a*∧*b*))∨(*b* ⊖ (*a*∧*b*)).

**Proposition 31 .**

Let *E* be a lattice ordered effect algebra. Then the following properties hold for every *a*, *b*, *c* ∈ *E*:

- *e*(*a*, *b*) = *e*(*b*, *a*),
- *e*(*a*, *b*) = 0 if and only if *a* = *b*,
- *e*(*a*, *b*) = *e*(*a*′, *b*′),
- *e*(*a*, *b*) ≤ *e*(*a*, *c*)∨*e*(*c*, *b*), if *c* ≤ *a*∧*b*.

**Proof**

(i) and (ii) are obvious. (iii): *e*(*a*, *b*) = (*a* ⊖ (*a*∧*b*))∨(*b* ⊖ (*a* ⊖ *b*)) = (*a*∨*b*)⊖(*a*∧*b*), *e*(*a*′, *b*′) = (*a*′∨*b*′) ⊖ (*a*∨*b*)′ = (*a*∧*b*)′ ⊖ (*a*∨*b*)′ = (1 ⊖ (*a*∧*b*))⊖(1 ⊖ (*a*∨*b*)), by Lemma 5, we have *e*(*a*′, *b*′) = (*a*∨*b*)⊖(*a*∧*b*). (iv): *e*(*a*, *b*) = (*a* ⊖ (*a*∧*b*))∨(*b* ⊖ (*a*∧*b*)) = (*a*∨*b*)⊖(*a*∧*b*), *e*(*a*, *c*) = (*a* ⊖ (*a*∧*c*))∨(*c* ⊖ (*a*∧*c*)) = (*a*∨*c*)⊖(*a*∧*c*), *e*(*b*, *c*) = (*b* ⊖ (*b*∧*c*))∨(*c* ⊖ (*b*∧*c*)) = (*b*∨*c*)⊖(*b*∧*c*); if *c* ≤ *a*∧*b*, we have *e*(*a*, *c*)∨*e*(*c*, *b*) ≥ *e*(*a*, *b*).

**Lemma 32 (Jenca and Pulmannová [9]).**

Let *I* be a Riesz ideal in a lattice ordered effect algebra *E*. Then *I* is a lattice ideal and ~_*I*_ is a lattice congruence. Moreover for all *a*, *b* ∈ *E*, *a* ~ _*I*_ *b* if and only if (*a* ⊖ (*a*∧*b*))∨(*b* ⊖ (*a*∧*b*)) ∈ *I*.

From the lemma, we have that if *I* is a Riesz ideal in a lattice ordered effect algebra *E*, then for all *a*, *b* ∈ *E*, *a* ~ _*I*_ *b* if and only if *e*(*a*, *b*) ∈ *I*.

**Definition 33 .**

Let *E* be an effect algebra and let *X* be a subset of *E*. Then *X* is called convex if for every *x*, *y* ∈ *X* and *z* ∈ *E*, *x* ≤ *z* ≤ *y* implies *z* ∈ *X*.

**Proposition 34 .**

Let *I* be a Riesz ideal of effect algebra *E*. Then [*a*]_*I*_ is convex for each *a* ∈ *E*.

**Proof**

Given *a* ∈ *E*, take *t*, *s* ∈ [*a*]_*I*_ with *t* ≤ *s* and let *t* ≤ *z* ≤ *s*. Since *z* ⊖ *t* ≤ *s* ⊖ *t* ~ _*I*_0, we have *z* ⊖ *t* ~ _*I*_0, too. Hence, *z* ~ _*I*_ *t*. That is *z* ∈ [*a*]_*I*_. So [*a*]_*I*_ is convex for each *a* ∈ *E*.

**Lemma 35 .**

Let *I* be a Riesz ideal of linear ordered effect algebra *E*. If *a* ≤ *b* and [*a*]_*I*_ ≠ [*b*]_*I*_, then for each *t* ∈ [*a*]_*I*_ and *s* ∈ [*b*]_*I*_, *t* ≤ *s*.

**Proof**

Suppose that there exist *t* ∈ [*a*]_*I*_ and *s* ∈ [*b*]_*I*_ such that *s* < *t*. We show that it is a contradiction. First, let *t* ≤ *b*. So we obtain *s* ≤ *t* ≤ *b* and by Proposition 34, *t* ∈ [*b*]_*I*_ which is a contradiction. Now let *b* ≤ *t*. Thus *a* ≤ *b* ≤ *t* and by Proposition 34, *b* ∈ [*a*]_*I*_ which is a contradiction again. Therefore for each *t* ∈ [*a*]_*I*_ and *s* ∈ [*b*]_*I*_, *t* ≤ *s*.

If *X* is a subset of lattice ordered effect algebra *E*, it is easy to check that for every *X*, *Y*⊆*E* if we have *X*⊆*Y*, then *X*′⊆*Y*′.

**Theorem 36 .**

Let *E* be a lattice ordered effect algebra and let *I* be a Riesz ideal of *E*. Let *X* be a subset of *E*. Then

- ${\left(\bar{X_{I}}\right)}^{\prime }=\bar{X_{I}^{\prime }}$,
- (*X*_*I*_^0^)′ = (*X*′)_*I*_^0^.

**Proof**

(i) Let $x\in {\left(\bar{X_{I}}\right)}^{\prime }$, so $x^{\prime }\in \bar{X_{I}}$ by Lemma 3 and *X*′ = {*x*′ : *x* ∈ *X*}. Therefore, [*x*′]_*I*_∩*X* ≠ *∅*. Hence there exists *y* ∈ [*x*′]_*I*_∩*X*. By Lemma 32 we have *e*(*y*, *x*′) ∈ *I* and *y* ∈ *X*. By Proposition 31, *e*(*y*′, *x*) ∈ *I* and *y*′ ∈ *X*′, so *y*′ ∈ [*x*]_*I*_∩*X*′ and this implies that $x\in \bar{X_{I}^{\prime }}$. Conversely, let $x\in \bar{X_{I}^{\prime }}$, so [*x*]_*I*_∩*X*′ ≠ *∅*. Suppose that *y* ∈ [*x*]_*I*_∩*X*′; then *y*′ ∈ [*x*′]_*I*_∩*X*. Similarly, $x\in {\left(\bar{X_{I}}\right)}^{\prime }$ and this proves part (i).

(ii) Let *x* ∈ (*X*_*I*_^0^)′, so *x*′ ∈ *X*_*I*_^0^ by Lemma 3. Therefore, [*x*′]_*I*_⊆*X*. Hence for each *y* ∈ [*x*′]_*I*_, we have *e*(*y*, *x*′) ∈ *I* and hence *e*(*y*′, *x*) ∈ *I* by Proposition 31. This implies that *y*′ ∈ [*x*]_*I*_⊆*X*′. So *x* ∈ (*X*′)_*I*_^0^. Conversely, let *x* ∈ (*X*′)_*I*_^0^, so [*x*]_*I*_⊆*X*′. Suppose that *y* ∈ [*x*]_*I*_⊆*X*′; then *y*′ ∈ [*x*′]_*I*_⊆*X* by Proposition 31. Hence *x*′ ∈ *X*_*I*_^0^. By Lemma 3 and *X*′ = {*x*′ : *x* ∈ *X*} again, we have *x* ∈ (*X*_*I*_^0^)′ and this proves part (ii).

**Theorem 37 .**

Let *E* be a lattice ordered effect algebra and let *X* be a subeffect algebra of *E*. Then

- $\bar{X_{I}}$ is a subeffect algebra of *E*,
- if *X* is an ideal and *I*⊆*X*, then *X*_*I*_^0^ is a subeffect algebra of *E*.

**Proof**

(i) Since 0,1 ∈ *X*, then $0,1\in \bar{X_{I}}$. For $a\in \bar{X_{I}}$, there exists *x* ∈ *X* such that *x* ∈ [*a*]_*I*_; then $x^{\prime }\in X\subseteq \bar{X_{I}}$. Since *x*′ ~ _*I*_ *a*′, so [*a*′]_*I*_∩*X* ≠ *∅*, therefore $a^{\prime }\in \bar{X_{I}}$. Let $a,b\in \bar{X_{I}}$ and *a*⊥*b*, then there are *x* ∈ [*a*]_*I*_, *y* ∈ [*b*]_*I*_, such that *x*, *y* ∈ *X* and *x*⊥*y*. Since *X* is subeffect algebra of *E*, we have *x* ⊕ *y* ∈ *X*. Note that *x* ~ _*I*_ *a* and *y* ~ _*I*_ *b*; we have *x* ⊕ *y* ~ _*I*_ *a* ⊕ *b* and hence *x* ⊕ *y* ∈ [*a* ⊕ *b*]_*I*_∩*X*. That is, $a⊕b\in \bar{X_{I}}$.

(ii) Let *I*⊆*X*. First we prove 0 ∈ *X*_*I*_^0^ and 1 ∈ *X*_*I*_^0^. For all *x* ∈ [0]_*I*_, by Definition 13, we have *x* ⊖ *i* = 0 ⊖ 0, *i* ≤ *x*, *i* ∈ *I*, thus *x* = *i* ∈ *I*⊆*X*, so [0]_*I*_⊆*X*, therefore 0 ∈ *X*_*I*_^0^. Similarly, 1 ∈ *X*_*I*_^0^ can be proved. Then we prove that *x* ∈ *X*_*I*_^0^ implies *x*′ ∈ *X*_*I*_^0^. For all *y* ∈ [*x*′]_*I*_, we have *y*′ ~ _*I*_ *x*. It follows that *y*′ ∈ *X*, then *y* ∈ *X*. Therefore *x*′ ∈ *X*_*I*_^0^. Let *x*, *y* ∈ *X*_*I*_^0^ and *x*⊥*y*. For all *z* ∈ [*x* ⊕ *y*]_*I*_, by Definition 13, we have *z* ⊖ *i* = (*x* ⊕ *y*) ⊖ *j* ≤ *x* ⊕ *y* ∈ *X*, *i* ≤ *z*, *j* ≤ *x* ⊕ *y*, *i* ∈ *I*. This yields that *z* ⊖ *i* ∈ *X*, thus *z* ∈ *X*. Therefore *x* ⊕ *y* ∈ *X*_*I*_^0^.

By the above theorem, we note that $\bar{I_{I}}$ and *I*_*I*_^0^ are subeffect algebras of *E*.

**Remark 38 .**

Let *E* be a lattice ordered effect algebra. Then

1. $\bar{{\left(E_{S}\right)}_{I}}$ is a subeffect algebra of *E*,
2. $\bar{C{\left(E\right)}_{I}}$ is a subeffect algebra of *E*.

We define a Boolean effect algebra to be an effect algebra *E* such that *E* = *C*(*E*) and an orthomodular effect algebra to be a lattice ordered effect algebra in which every element is principal.

**Proposition 39 .**

Let *I* be a Riesz ideal of a linear ordered effect algebra *E* and let *X* be a convex subset of *E*. Then *X*_*I*_^0^ and $\bar{X_{I}}$ are convex.

**Proof**

Let *x*, *y* ∈ *X*_*I*_^0^ and *x* ≤ *z* ≤ *y*. We divide the proof into three cases. First let *x*~_*I*_ *y*, we have *x*, *y* ∈ [*a*]_*I*_ for some *a* ∈ *E*. By Proposition 34, [*a*]_*I*_ is convex, so that *z* ∈ [*a*]_*I*_. It follows that [*z*]_*I*_ = [*a*]_*I*_ = [*x*]_*I*_⊆*X*; therefore *z* ∈ *X*_*I*_^0^. Next let [*x*]_*I*_ ≠ [*y*]_*I*_ and *z* ∈ [*x*]_*I*_. Then [*z*]_*I*_ = [*x*]_*I*_. Since *x* ∈ *X*_*I*_^0^, [*z*]_*I*_ = [*x*]_*I*_⊆*X*. So *z* ∈ *X*_*I*_^0^. Similarly for [*x*]_*I*_ ≠ [*y*]_*I*_ and *z* ∈ [*y*]_*I*_, we can prove that *z* ∈ *X*_*I*_^0^ and we omit it. Finally let [*x*]_*I*_ ≠ [*y*]_*I*_, $z\underline{\in }{\left[x\right]}_{I}$, and $z\underline{\in }{\left[y\right]}_{I}$. Now we show [*z*]_*I*_⊆*X*. So let *t* ∈ [*z*]_*I*_. By Lemma 35, for all *u* ∈ [*x*]_*I*_ and *v* ∈ [*y*]_*I*_ we have *u* ≤ *t* ≤ *v*. Since [*x*]_*I*_, [*y*]_*I*_⊆*X*, we have *u*, *v* ∈ *X*. Note that *X* is convex; we obtain *t* ∈ *X* and hence [*z*]_*I*_⊆*X*. It follows that *z* ∈ *X*_*I*_^0^. This shows that *X*_*I*_^0^ is convex.

Now, let $x,y\in \bar{X_{I}}$ and *x* ≤ *z* ≤ *y*. We divide the proof into three cases. First let *x*~_*I*_ *y*; we have *x*, *y* ∈ [*a*]_*I*_ for some *a* ∈ *E*. By Proposition 34, [*a*]_*I*_ is convex, so that *z* ∈ [*a*]_*I*_. It follows that [*z*]_*I*_ = [*a*]_*I*_ = [*x*]_*I*_ and [*x*]_*I*_∩*X* ≠ *∅*; therefore [*z*]_*I*_∩*X* ≠ *∅*. Hence $z\in \bar{X_{I}}$. Next let [*x*]_*I*_ ≠ [*y*]_*I*_ and *z* ∈ [*x*]_*I*_. Then [*z*]_*I*_ = [*x*]_*I*_. Since $x\in \bar{X_{I}}$, then [*x*]_*I*_∩*X* ≠ *∅* and hence [*z*]_*I*_∩*X* ≠ *∅*. Therefore $z\in \bar{X_{I}}$. Similarly for [*x*]_*I*_ ≠ [*y*]_*I*_ and *z* ∈ [*y*]_*I*_, we can prove that $z\in \bar{X_{I}}$. Now let [*x*]_*I*_ ≠ [*y*]_*I*_, $z\bar{\in }{\left[x\right]}_{I}$, and $z\bar{\in }{\left[y\right]}_{I}$. We must show [*z*]_*I*_∩*X* ≠ *∅*. Since $x,y\in \bar{X_{I}}$, we have [*x*]_*I*_∩*X* ≠ *∅* and [*y*]_*I*_∩*X* ≠ *∅*. So there exist *u* ∈ [*x*]_*I*_∩*X* and *v* ∈ [*y*]_*I*_∩*X*. By Lemma 35, we have *u* ≤ *z* ≤ *v*. So *z* ∈ *X* since *X* is convex. This implies that [*z*]_*I*_∩*X* ≠ *∅* and hence $\bar{X_{I}}$ is convex.

**Proposition 40 .**

Let *E* be a lattice ordered effect algebra, *I* and *J* two Riesz ideals of *E*, and *X* a subset of *E*.

- If *X* is an ideal of *E* and *I*, *J*⊆*X*, then *X*_*I*_^0^∩*X*_*J*_^0^ = *X*_*I*∩*J*_^0^,
- If *X* is crisp with respect to *I* or *J*, then $\bar{X_{I\cap J}}=\bar{X_{I}}\cap \bar{X_{J}}$.

**Proof**

(i) By Lemma 29, *X*_*I*_^0^∩*X*_*J*_^0^⊆*X*_*I*∩*J*_^0^. Conversely, assume *x* ∈ *X*_*I*∩*J*_^0^. Then [*x*]_*I*∩*J*_⊆*X* and hence *x* ∈ *X*. Now, let *y* ∈ [*x*]_*I*_, so *e*(*x*, *y*) ∈ *I*⊆*X*. Because *X* is an ideal and *x* ∈ *X*, so *x*∧*y* ∈ *X*. Hence we have *e*(*x*, *y*)⊕(*x*∧*y*) ∈ *X*. On the other hand, by Lemma 6, we have *e*(*x*, *y*) = (*x*∨*y*)⊖(*x*∧*y*) and so *y* ≤ *x*∨*y* = *e*(*x*, *y*)⊕(*x*∧*y*). This means that *y* ∈ *X*, which implies *x* ∈ *X*_*I*_^0^. Similarly, we can obtain *x* ∈ *X*_*J*_^0^ and so *x* ∈ *X*_*I*_^0^∩*X*_*J*_^0^. Therefore *X*_*I*_^0^∩*X*_*J*_^0^ = *X*_*I*∩*J*_^0^.

(ii) First, assume that *X* is crisp with respect to *I*, so $\bar{X_{I}}=X$. Therefore, we have $\bar{X_{I}}\cap \bar{X_{J}}=X\cap \bar{X_{I}}=X\subseteq \bar{X_{I\cap J}}$. Also by Lemma 29, $\bar{X_{I\cap J}}\subseteq \bar{X_{I}}\cap \bar{X_{J}}$, and it proves the theorem.

## 5. Approximation in Orthomodular Effect Algebras and Boolean Effect Algebras

We define a Boolean effect algebra to be an effect algebra *E* such that *E* = *C*(*E*) and an orthomodular effect algebra to be a lattice ordered effect algebra in which every element is principal.

Let *X* and *Y* be subsets of *E*. We define *X*∨*Y* = {*a*∨*b* : *a* ∈ *X*, *b* ∈ *Y*} and define the downset *X* ^↓^ = {*c* ∈ *E* : ∃*a* ∈ *X*, *c* ≤ *a*}.

**Proposition 41 .**

Let *E* be an orthomodular effect algebra and let *I* be a Riesz ideal of *E*, and let *X*, *Y* be subsets of *E*. Then $\bar{{\left({\left(X\vee Y\right)}^{↓}\right)}_{I}}\subseteq {\left(\bar{X_{I}}\vee \bar{Y_{I}}\right)}^{↓}$.

**Proof**

Let $a\in \bar{{\left({\left(X\vee Y\right)}^{↓}\right)}_{I}}$, so [*a*]_*I*_∩(*X*∨*Y*)^↓^ ≠ *∅*. Thus there exists *b* ∈ [*a*]_*I*_∩(*X*∨*Y*)^↓^ and consequently there exist *x* ∈ *X* and *y* ∈ *Y* such that *b* ≤ *x*∨*y*. On the other hand, since [*a*]_*I*_ = [*b*]_*I*_, by Definition 13, there are *i*, *j* ∈ *I*, *i* ≤ *a*, *j* ≤ *b* such that *a* = (*b* ⊖ *j*) ⊕ *i* ≤ *b*∨*i* ≤ *x*∨*y*∨*i* = (*x*∨*i*)∨*y*. By Lemma 32, [*x*∨*i*]_*I*_ = [*x*]_*I*_, $x\in \bar{X_{I}}$ and $y\in \bar{Y_{I}}$, hence $a\in {\left(\bar{X_{I}}\vee \bar{Y_{I}}\right)}^{↓}$.

**Proposition 42 .**

Let *E* be an orthomodular effect algebra and let *I* be a Riesz ideal of *E*, and let *X*, *Y* be subsets of *E*. Then (*X*_*I*_^0^∨*Y*_*I*_^0^)^↓^⊆((*X*∨*Y*)^↓^)_*I*_^0^.

**Proof**

Suppose that *a* ∈ (*X*_*I*_^0^∨*Y*_*I*_^0^)^↓^, so there exist *x* ∈ *X*_*I*_^0^ and *y* ∈ *Y*_*I*_^0^ such that *a* ≤ *x*∨*y*. Now, let *b* ∈ [*a*]_*I*_; thus by by Definition 13, there are *i*, *j* ∈ *I*, *i* ≤ *a*, *j* ≤ *b* such that *b* = (*a* ⊖ *i*) ⊕ *j* ≤ *a*∨*j* ≤ *x*∨*y*∨*j* = (*x*∨*j*)∨*y*. By Lemma 32, we have *x*∨*j* ∈ [*x*∨*j*]_*I*_ = [*x*]_*I*_⊆*X* and *y* ∈ *Y*. Hence *b* ∈ *X*∨*Y*, this implies that *a* ∈ ((*X*∨*Y*)^↓^)_*I*_^0^.

The following example shows that we can not replace the inclusion symbol ⊆ by equal sign in Proposition 42.

**Example 43 .**

Consider *E* = {0, *a*, *b*, *c*, *d*, *e*, 1}, as the lattice-ordered effect algebra in Example 26. Let *X* = {0}, *Y* = {0, *b*, *c*, 1} be the subsets of *E* and let *I* = {0, *a*} be the Riesz ideal of *E*. We have (*X*∨*Y*)^↓^ = *E*, *X*_*I*_^0^ = *∅* and *Y*_*I*_^0^ = {*b*, *c*}. Therefore ((*X*∨*Y*)^↓^)_*I*_^0^ = *E* and (*X*_*I*_^0^∨*Y*_*I*_^0^)^↓^ = *∅*. This shows that (*X*_*I*_^0^∨*Y*_*I*_^0^)^↓^ ≠ ((*X*∨*Y*)^↓^)_*I*_^0^.

In what follows, that is, Propositions 44, 45, 47, 49, Examples 46 and 51, and Corollary 48, *E* is a Boolean effect algebra.

**Proposition 44 .**

Let *E* be a Boolean effect algebra and let *I*, *J* be two Riesz ideals of *E*. Then (*I*∨*J*)^↓^ is also a Riesz ideal of *E*.

**Proof**

Let *a*, *b* ∈ (*I*∨*J*)^↓^ and *a*⊥*b*; then *a* ≤ *x*_1_∨*y*_1_, *b* ≤ *x*_2_∨*y*_2_, where *x*_*i*_ ∈ *I*, *y*_*i*_ ∈ *J*, *i* = 1,2. Since *a* ⊕ *b* = *a*∨*b*, then *a* ⊕ *b* ≤ (*x*_1_∨*y*_1_)∨(*x*_2_∨*y*_2_) = (*x*_1_∨*x*_2_)∨(*y*_1_∨*y*_2_)∈(*I*∨*J*)^↓^. So (*I*∨*J*)^↓^ is an ideal. Let *x* ∈ (*I*∨*J*)^↓^; then *x* ≤ *i*∨*j*, where *i* ∈ *I*, *j* ∈ *J*. For *a*, *b* ∈ *E*, *a*⊥*b* and *x* ≤ *a* ⊕ *b*, we have *x* ≤ (*a*∨*b*)∧(*i*∨*j*). Taking *a*_1_ = *a*∧(*i*∨*j*) = (*a*∧*i*)∨(*a*∧*j*), *b*_1_ = (*b*∧*i*)∨(*b*∧*j*), we have *a*_1_, *b*_1_ ∈ (*I*∨*J*)^↓^ and *a*_1_ ≤ *a*, *b*_1_ ≤ *b*. Since *E* is a distributive lattice ordered effect algebra and (*E*, ≤) is an orthomodular lattice, it follows that *a*_1_ ⊕ *b*_1_ = (*a*∧(*i*∨*j*))⊕(*b*∧(*i*∨*j*)) = (*a*∧(*i*∨*j*))∨(*b*∧(*i*∨*j*)) = (*a*∨*b*)∧(*i*∨*j*). Therefore, *x* ≤ *a*_1_ ⊕ *b*_1_. Hence, (*I*∨*J*)^↓^ is a Riesz ideal.

**Proposition 45 .**

Let *E* be a Boolean effect algebra, and let *I*, *J* be two Riesz ideals of *E* and let *X* be a subset of *E*. Then $\bar{X_{{\left(I\vee J\right)}^{↓}}}\subseteq {\left(\bar{X_{I}}\vee \bar{X_{J}}\right)}^{↓}$.

**Proof**

Assume that *X* is a subset of *E*. Let $x\in \bar{X_{{\left(I\vee J\right)}^{↓}}}$, so [*x*]_(*I*∨*J*)^↓^_∩*X* ≠ *∅*. Thus there exists *s* ∈ [*x*]_(*I*∨*J*)^↓^_∩*X* which implies *e*(*s*, *x*)∈(*I*∨*J*)^↓^ and *s* ∈ *X*; hence by Definition 13, there exist *h*, *k* ∈ (*I*∨*J*)^↓^, *h* ≤ *s*, *k* ≤ *x*, such that *x* = (*s* ⊖ *h*) ⊕ *k* and also there exist *i* ∈ *I*, *j* ∈ *J* such that *k* ≤ *i*∨*j*. Then *x* = (*s* ⊖ *h*) ⊕ *k* ≤ *s*∨*k* ≤ *s*∨(*i*∨*j*) = (*s*∨*i*)∨(*s*∨*j*). On the other hand by Lemma 32, we have [*s*∨*i*]_*I*_ = [*s*]_*I*_ and [*s*∨*j*]_*J*_ = [*s*]_*J*_. Therefore, $x\in {\left(\bar{X_{I}}\vee \bar{X_{J}}\right)}^{↓}$.

The following example shows that the symbol inclusion in Proposition 45 can be proper.

**Example 46 .**

Consider *E* = {0, *a*, *b*, 1} as the Boolean effect algebra in Example 25. It is easy to check that *I* = {0, *a*}, *J* = {0} are two ideals of *E*. And obviously, (*I*∨*J*)^↓^ = {0, *a*} is an ideal of *E* too. We have [0]_*I*_ = *I*, [*a*]_*I*_ = *I*, [*b*]_*I*_ = {1, *b*}, [1]_*I*_ = {*b*, 1}, [0]_*J*_ = {0}, [*a*]_*J*_ = {*a*}, [*b*]_*J*_ = {*b*}, [1]_*J*_ = {1}. Assume *X* = {*b*}; then $\bar{X_{{\left(I\vee J\right)}^{↓}}}=\{0,a\}$, $\bar{X_{I}}=\{0,a\}$, $\bar{X_{J}}=\{b\}$, ${\left(\bar{X_{I}}\vee \bar{X_{J}}\right)}^{↓}=E$. So $\bar{X_{{\left(I\vee J\right)}^{↓}}}\subset {\left(\bar{X_{I}}\vee \bar{X_{J}}\right)}^{↓}$.

**Proposition 47 .**

Let *E* be a Boolean effect algebra, *I*, *J* two Riesz ideals of *E*, and *X* a subset of *E*. Then *X*_(*I*∨*J*)^↓^_^0^⊆(*X*_*I*_^0^∨*X*_*J*_^0^)^↓^. Furthermore, if *a* ∈ (*X*_*I*_^0^∨*X*_*J*_^0^)^↓^, then one obtains [*a*]_(*I*∨*J*)^↓^_⊆(*X*_*I*_^0^∨*X*_*J*_^0^)^↓^.

**Proof**

Let *a* ∈ *X*_(*I*∨*J*)^↓^_^0^, so [*a*]_(*I*∨*J*)^↓^_⊆*X*. Since *a* ≤ *a*∨*a* and [*a*]_*I*_, [*a*]_*J*_⊆[*a*]_(*I*∨*J*)^↓^_, hence *a* ∈ (*X*_*I*_^0^∨*X*_*J*_^0^)^↓^.

Now, suppose that *a* ∈ (*X*_*I*_^0^∨*X*_*J*_^0^)^↓^. There exist *x*_1_ ∈ *X*_*I*_^0^ and *x*_2_ ∈ *X*_*J*_^0^ such that *a* ≤ *x*_1_∨*x*_2_. Suppose that *b* ∈ [*a*]_(*I*∨*J*)^↓^_, so there exist *i*, *j* ∈ (*I*∨*J*)^↓^, *i* ≤ *a*, *j* ≤ *b* such that *b* = (*a* ⊖ *i*) ⊕ *j*. Also, there are *h* ∈ *I* and *k* ∈ *J* such that *j* ≤ *h*∨*k*. Then *b* ≤ *a*∨*j* ≤ *x*_1_∨*x*_2_∨*h*∨*k* ≤ (*x*_1_∨*h*)∨(*x*_2_∨*k*). By Lemma 32, we have [*x*_1_∨*h*]_*I*_ = [*x*_1_]_*I*_ and [*x*_2_∨*k*]_*I*_ = [*x*_2_]_*I*_. This implies that [*a*]_(*I*∨*J*)^↓^_⊆(*X*_*I*_^0^∨*X*_*J*_^0^)^↓^.

From the above proposition, we have the following corollary.

**Corollary 48 .**

Let *E* be a Boolean effect algebra, and let *I*, *J* be two Riesz ideals of *E* and let *X* be a lattice ideal of *E*. Then one has *X*_(*I*∨*J*)^↓^_^0^ = (*X*_*I*_^0^∨*X*_*J*_^0^)^↓^.

**Proof**

By Proposition 47, we have *X*_(*I*∨*J*)^↓^_^0^⊆(*X*_*I*_^0^∨*X*_*J*_^0^)^↓^. Since *X* is an ideal, we can show easily, (*X*_*I*_^0^∨*X*_*J*_^0^)^↓^⊆*X*. Assume *a* ∈ (*X*_*I*_^0^∨*X*_*J*_^0^)^↓^, so [*a*]_(*I*∨*J*)^↓^_⊆(*X*_*I*_^0^∨*X*_*J*_^0^)^↓^⊆*X* from Proposition 47. That is, *a* ∈ *X*_(*I*∨*J*)^↓^_^0^. It yields that (*X*_*I*_^0^∨*X*_*J*_^0^)^↓^⊆*X*_(*I*∨*J*)^↓^_^0^. Therefore, *X*_(*I*∨*J*)^↓^_^0^ = (*X*_*I*_^0^∨*X*_*J*_^0^)^↓^.

**Proposition 49 .**

Let *E* be a lattice ordered effect algebra and *F* a Boolean effect algebra. If *I* is a Riesz ideal of *E* and *h* : *E* → *F* is a monomorphism, then *h*(*I*) is a Riesz ideal of *F*.

**Proof**

First, we prove that *h*(*I*) is an ideal. Let *h*(*x*), *h*(*y*) ∈ *h*(*I*) and *h*(*x*)⊥*h*(*y*), where *x*, *y* ∈ *I*. Since *h* is a monomorphism, then *h*(*x*) ⊕ *h*(*y*) = *h*(*x* ⊕ *y*) ∈ *h*(*I*). So *h*(*I*) is an ideal of *F*. Next, we prove that *h*(*I*) is a Riesz ideal. For any *h*(*x*) ∈ *h*(*I*), let *h*(*x*) ≤ *a* ⊕ *b*, *a*, *b* ∈ *F*, *a*⊥*b*. Take *a*_1_ = *h*(*x*)∧*a*, *b*_1_ = *h*(*x*)∧*b*; then *a*_1_, *b*_1_ ∈ *h*(*I*) and *a*_1_ ≤ *a*, *b*_1_ ≤ *b*. Since *F* is a distributive lattice ordered effect algebra, and (*F*, ≤) is an orthomodular lattice, it follows that *a*_1_ ⊕ *b*_1_ = (*h*(*x*)∧*a*)⊕(*h*(*x*)∧*b*) = *h*(*x*)∧(*a*∨*b*). Therefore, *h*(*x*) ≤ *a*_1_ ⊕ *b*_1_. Hence, *h*(*I*) is a Riesz ideal of *F*.

**Theorem 50 .**

Let *E*, *F* be lattice ordered effect algebras, let *h* : *E* → *F* be a morphism, and let *I*_*h*_ : = {*a* ∈ *E* : *h*(*a*) = 0} be the kernel of *h*.

1. If *X* is a subset of *E*, then $h(\bar{X_{I_{h}}})=h(X)$.
2. Assume that *h* is a monomorphism and (*F*, ≤) is a Boolean effect algebra. If *X* is a subset of *E* and *I* is a Riesz ideal of *E* containing *I*_*h*_, then $h(\bar{X_{I}})=\bar{h{\left(X\right)}_{h(I)}}$.

**Proof**

(1) Since $X\subseteq \bar{X_{I_{h}}}$, it follows that $h(X)\subseteq h(\bar{X_{I_{h}}})$. Conversely, let $y\in h(\bar{X_{I_{h}}})$; then there exists $x\in \bar{X_{I_{h}}}$ such that *h*(*x*) = *y*. So we have [*x*]_*I*_*h*__∩*X* ≠ *∅*. Let *a* ∈ [*x*]_*I*_*h*__∩*X*. We have *a* ∈ [*x*]_*I*_*h*__ and *a* ∈ *X*, so there exist *i*, *j* ∈ *I*_*h*_, *i* ≤ *a*, *j* ≤ *x* such that *a* = (*x* ⊖ *j*) ⊕ *i*. Since *h* is a morphism, we get that *h*(*a*) = *h*(*x* ⊖ *j*) ⊕ *h*(*i*) = *h*(*x*) ⊖ *h*(*j*) ⊕ *h*(*i*) = *h*(*x*). It follows that *h*(*a*) = *y* ∈ *h*(*X*), which proves the theorem.

(2) Let $y\in h(\bar{X_{I}})$. Then there exists $x\in \bar{X_{I}}$ such that *y* = *h*(*x*). Since [*x*]_*I*_∩*X* ≠ *∅*, suppose *z* ∈ [*x*]_*I*_∩*X*, so we have [*z*]_*I*_ = [*x*]_*I*_ and *z* ∈ *X*. Hence there exist *i*, *j* ∈ *I*, *i* ≤ *z*, *j* ≤ *x* such that *z* = (*x* ⊖ *j*) ⊕ *i*. Since *h* a monomorphism, then [*h*(*z*)]_*h*(*I*)_ = [*h*(*x*)]_*h*(*I*)_ and *h*(*z*) ∈ *h*(*X*). Therefore, $y\in \bar{h{\left(X\right)}_{h(I)}}$.

Conversely, let $y\in \bar{h{\left(X\right)}_{h(I)}}$, so [*y*]_*h*(*I*)_∩*h*(*X*) ≠ *∅*. Suppose *z* ∈ [*y*]_*h*(*I*)_∩*h*(*X*); it implies that [*y*]_*h*(*I*)_ = [*z*]_*h*(*I*)_ and *z* ∈ *h*(*X*). Since *h* is a monomorphism, there exists *x* ∈ *E* such that *y* = *h*(*x*) and there exist *t* ∈ *X* and *s* ∈ *I* such that *z* = *h*(*t*) and *e*(*h*(*t*), *h*(*x*)) = *h*(*s*), so *h*(*e*(*t*, *x*)) = *h*(*s*) and it implies that *h*(*e*(*t*, *x*) ⊖ *s*) = 0. Hence *e*(*t*, *x*) ⊖ *s* ∈ *I*. So *e*(*t*, *x*) ∈ *I* and *t* ∈ [*x*]_*I*_ by Lemma 32. Now, we conclude that [*x*]_*I*_∩*X* ≠ *∅*, so $x\in \bar{X_{I}}$. This proves the theorem.

In order to well illustrate the above theorem, we give the following example.

**Example 51 .**

Consider *E* = {0, *a*, *b*, 1}, as the lattice ordered effect algebra in Example 25. Also, let *F* = {0, *y*_1_, *y*_2_, 1}(0 < *y*_1_, *y*_2_ < 1). For all *y* ∈ *F*, define *y*_1_ ⊕ *y*_2_ = *y*_2_ ⊕ *y*_1_ = 1, *y* ⊕ 0 = 0 ⊕ *y* = *y*; otherwise ⊕ is undefined. Then (*F*, ⊕, 0,1) is a Boolean effect algebra.

The map *ϕ* : *E* → *F* is given by *ϕ*(1) = 1, *ϕ*(*a*) = *y*_1_, *ϕ*(*b*) = *y*_2_, *ϕ*(0) = 0. We can easily check that *ϕ* is a monomorphism. Since *ker*⁡*ϕ* = {0}, so [0]_*ker*⁡*ϕ*_ = {1}, [*a*]_*ker*⁡*ϕ*_ = {*a*}, [*b*]_*ker*⁡*ϕ*_ = {*b*}, [1]_*ker*⁡*ϕ*_ = {1}. Suppose that *X* = {*a*, *b*}; then *ϕ*(*X*) = {*y*_1_, *y*_2_} and $\phi (\bar{X_{\ker\phi }})=\phi (X)$.

## 6. Conclusion

The earliest research on rough set theory of algebraic structures are on semigroups, groups, and modules. We set up the rough structure on partial algebraic system. The core foundation of rough set theory and application is a pair of approximation operators induced from the approximation space, namely, the closure operator and interior operator (also called upper and lower approximation set). We try to define rough approximation operators by a congruence relation on effect algebras and thus induce rough structure of effect algebras. Because effect algebras are incomplete algebras, not any two elements can compute, so we give a full operation on an effect algebra to construct a distance function. Moreover by use of Riesz ideals and distance functions we induced a congruence relation and then we obtain rough approximation operators and rough effect algebra system.

## Figures and Tables

**Figure 1:**